# The Prognostic Value of Serum Calcium Levels in Elderly Dilated Cardiomyopathy Patients

**DOI:** 10.5334/gh.1304

**Published:** 2024-02-28

**Authors:** Xinyi Li, Wenfei He, Qiqi Song, Qingshan Ding, Xiaonan Zhang, Zhigang Zeng, Weiping Deng, Gang Deng, Lichang Guan, Wanzi Hong, Yaoxin Liu, Fen Shu, Lishu Xu, Ning Tan, Jinjin Ma, Lei Jiang

**Affiliations:** 1 School of Medicine, South China University of Technology, Guangzhou 510006, China; 2 Department of Cardiology, Guangdong Cardiovascular Institute, Guangdong Provincial People’s Hospital (Guangdong Academy of Medical Sciences), Southern Medical University, Guangzhou 510080, China; 3 Department of Cardiology, Guangdong Provincial People’s Hospital’s Nanhai Hospital, The Second People’s Hospital of Nanhai District, Foshan 528000, China

**Keywords:** Biomarkers, Clinical study, Diagnostic advances, Geriatric cardiomyopathy

## Abstract

**Background:**

It is unclear whether serum calcium on admission is associated with clinical outcomes in dilated cardiomyopathy (DCM). In this study, we conducted a retrospective study spanning a decade to investigate the prognostic value of baseline calcium in elderly patients with DCM.

**Methods:**

A total of 1,089 consecutive elderly patients (age ≥60 years) diagnosed with DCM were retrospectively enrolled from January 2010 to December 2019. Univariate and multivariate analyses were performed to investigate the association of serum calcium with their clinical outcomes.

**Results:**

In this study, the average age of the subjects was 68.36 ± 6.31 years. Receiver operating characteristic (ROC) curve analysis showed that serum calcium level had a great sensitivity and specificity for predicting in-hospital death, with an AUC of 0.732. Kaplan–Meier survival analysis showed that patients with a serum calcium >8.62 mg/dL had a better prognosis than those with a serum calcium ≤8.62 mg/dL (log-rank χ^2^ 40.84, p < 0.001). After adjusting for several common risk factors, a serum calcium ≤8.62 mg/dL was related to a higher risk of long-term mortality (HR: 1.449; 95% CI: 1.115~1.882; p = 0.005).

**Conclusions:**

Serum calcium level could be served as a simple and affordable tool to evaluate patients’ prognosis in DCM.

## Introduction

The general definition of dilated cardiomyopathy (DCM) is the presence of left ventricular (LV) or biventricular dilatation and systolic dysfunction (LVEF <45%) unexplained solely by abnormal loading conditions (e.g., hypertension, valve disease) or coronary artery disease [1,2]. Due to refractory heart failure (HF) and sudden cardiac death (SCD), patients with advanced DCM need cardiac transplantation. The overall management of DCM places a heavy financial burden on global health care systems, which costs $4–10 billion/year in the United States alone [3,4,5]. The therapy with angiotensin-converting enzyme inhibitors or angiotensin II-receptor blockers (ACEIs/ARBs) and β-blockers has been considered as optimal medical treatment (OMT) at present, however, such patients still associated with an approximately ten-year mortality rate of 40% despite receiving OMT [6,7]. The mortality of elderly patients should be higher because of their more complicated heath condition, who are more likely to benefit from prognostic information.

In recent years, scientists have emphasized the prognostic benefits of randomized controlled studies in the real world and the importance of long-term follow-up. Patients with DCM present myocardial systolic dysfunction and calcium ions play a key role in the excitation and contraction of cardiac muscle fibers [2,8], which suggests some relationship between calcium ions and the progression of DCM. Previous studies have demonstrated that hypocalcemia is associated with poor clinical outcomes in cardiovascular disease [9,10,11]. Based on this evidence, we speculated that serum calcium could serve as a prognostic indicator in DCM.

## Materials and Methods

### Study population

For this study, we retrospectively investigated 1,095 consecutive elderly patients (age ≥60 years [12,13]) admitted for DCM in our hospital from January 2010 to December 2019. Dilated cardiomyopathy is defined by the scientific statement established by the European Society of Cardiology (ESC) [2]. Serum calcium data were missing in six patients and 1,089 patients were enrolled. This study was approved by the Ethics Committee of Guangdong Provincial People’s Hospital with a waiver of written informed consent. Oral informed consent was obtained from conscious patients and all vulnerable patients’ guardian/next of kin by telephone and recorded by trained nurses during the follow-up period.

### Data source

Baseline characteristics, medical history, and laboratory results were collected from the electronic medical database. Clinical information was collected from an electronic case report form by one researcher and independently confirmed by another researcher. Basal serum calcium samples were collected on the following morning after admission and measured by spectrophotometric method. The LVEF was determined using Simpson’s biplane method and linear internal measurements of the LV and its walls were performed in the parasternal view.

### Definition and endpoints

Lower serum calcium on admission was defined as a serum calcium level of less than 8.4 mg/dL according to the standards established by laboratory. During the follow-up, the primary endpoint was in-hospital mortality, and the secondary endpoints were long-term mortality and major adverse clinical events (MACEs) which included acute HF, malignant arrhythmia but not vascular diseases.

### Statistical analysis

Continuous variables are presented as the mean ± SD and compared using Student’s t-test for parametric variables, the Mann–Whitney U-test for non-parametric variables. Categorical variables are expressed as the number and percentage and compared using Pearson’s chisquare tests. Kaplan–Meier survival curves are drawn to compare cumulative event rates between groups by the log-rank test. To assess the prognostic value of serum calcium, receiver operator characteristic (ROC) curves are drawn to determine the cutoff values. Logistic regression analyses and Cox proportional hazard regression models are conducted to evaluate the association of serum calcium with prognosis, in which the adjusted odds ratio (OR), hazard ratio (HR) and 95% confidence interval (CI) were calculated. Data were analyzed statistically using SPSS software version 26.0 (IBM Corp., Armonk, New York, USA). A double-sided p-value < 0.05 denoted statistical significance.

## Results

A total of 1,089 patients met the inclusion criteria and were divided into two groups based on their serum calcium levels (943 with normal calcium, 146 with lower calcium). There were 160 (14.7%) patients with New York Heart Association (NYHA) grade IV disease and 24 patients with tumor history (not active tumor) but none with nephrotic syndrome. Forty-five individuals died in hospital and a larger percentage showed in patients with lower calcium (11.6% vs. 3.0%). During hospitalization, 274 patients occurred atrial fibrillation but no significant difference between groups (27.4% in lower group and 24.8% in normal group). During a median follow-up of 67 ± 1.8 months, a total of 1,014 patients were successfully followed up and 461 all-cause deaths (303 males, 158 females) were recorded, including 371 (41.7%) with normal calcium and 90 (72.0%) with lower calcium.

The medical history of smoking, hypertension, and diabetes were similar between the two groups, whereas patients with normal calcium were younger and had fewer men. Besides, patients with lower calcium had a higher value of serum creatinine and lower value of high-density lipoprotein cholesterol (HDL-C), but the LVEF presented no statistical difference between groups (Table 1).

**Table 1:** Baseline characteristics between normal and Lower calcium group.


VARIABLES	PATIENTS WITH DCM (N = 1089)		p-VALUE

	NORMAL CALCIUM	LOWER CALCIUM	

	(n = 943)	(n = 146)	

Demographic			

Age, y	68.12 ± 6.15	69.94 ± 7.08	0.005

Male, n(%)	601(63.7)	111(76.0)	0.004

Medical History			

Smoking history, n(%)	244(25.9)	45(30.8)	0.208

Hypertension, n(%)	309(32.8)	44(30.1)	0.527

Diabetes, n(%)	228(24.2)	34(23.3)	0.815

Parameters and medications			

Serum chloride, mmol/L	103.27 ± 5.03	101.41 ± 6.54	0.001

Serum sodium, mmol/L	138.24 ± 3.75	136.76 ± 12.46	0.090

Serum potassium, mmol/L	3.8 ± 0.51	3.76 ± 0.75	0.562

WBC count, 10^9^/L	7.5 ± 2.82	7.51 ± 2.68	0.967

Neutrophil count, 10^9^/L	5.03 ± 2.57	4.96 ± 2.59	0.429

Lymphocyte count, 10^9^/L	1.64 ± 1.16	1.67 ± 0.68	0.453

Hemoglobin, g/L	131.14 ± 18.88	131.92 ± 17.93	0.641

Glucose, mmol/L	6.83 ± 2.99	7.00 ± 3.14	0.496

CREA, umol/L	107.18 ± 67.58	145.76 ± 103.08	<0.001

Uric, umol/L	501.68 ± 169.75	519.53 ± 236.16	0.447

CHOL, mmol/L	4.43 ± 1.08	3.90 ± 1.09	<0.001

LDL-C, mmol/L	2.79 ± 0.85	2.41 ± 0.88	<0.001

HDL-C, mmol/L	1.05 ± 0.30	0.89 ± 0.35	<0.001

TBIL, umol/L	21.60 ± 15.70	28.7 ± 47.69	0.002

LVEF, %	38.58 ± 16.12	36.70 ± 17.56	0.235

Diuretics use, n(%)	850(90.1)	129(88.4)	0.506

Digoxin use, n(%)	436(46.2)	80(54.8)	0.054

Clinical outcomes			

In-hospital mortality, n(%)	28(3.0)	17(11.6)	<0.001

Long-term mortality, n(%)	371(41.7)	90(72.0)	<0.001


**Abbreviations:** DCM: dilated cardiomyopathy; WBC: white blood cell; CREA: creatinine; CHOL: total cholesterol; LDL-C: low density lipoprotein cholesterol; HDL-C: high density lipoprotein cholesterol; TBIL: total bilirubin; LVEF: left ventricle ejection fraction.

**Figure 1:**
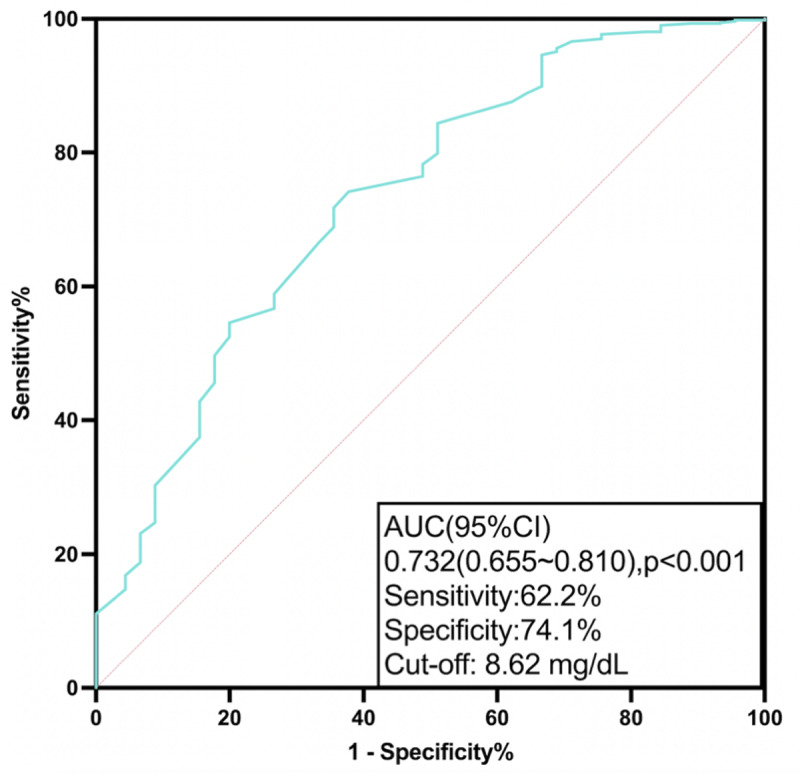
ROC curve of serum calcium in predicting in-hospital mortality.

On the one hand, univariate logistic regression analysis showed that serum calcium was inversely associated with in-hospital mortality (OR: 0.204; 95% CI: 0.121 to 0.345; p < 0.001). After adjusting for other significant indicators, serum calcium remained independently related to in-hospital death (OR: 0.340; 95% CI: 0.160~0.720; p = 0.005) (Table 2). The result of collinearity diagnostics showed that there were no multicollinearity issues among these variables (Variance inflation factor, VIF < 10). Receiver operating characteristic curve analysis demonstrated that serum calcium had great predictive power for in-hospital mortality (AUC = 0.732, 95% CI: 0.655~0.810; p < 0.001), in which the optimal cutoff value was 8.62 mg/dL, with a sensitivity of 62.2% and specificity of 74.1% (Figure 1).

**Table 2:** Logistic regression analyses for in-hospital mortality.


VARIABLES	UNIVARIATE ANALYSIS		MULTIVARIATE ANALYSIS	
	
	OR	p-VALUE	OR	p-VALUE

Serum calcium	0.204 (0.121~0.345)	<0.001	0.340 (0.160~0.720)	0.005

Age	1.048 (1.003~1.095)	0.038	0.980 (0.922~1.042)	0.525

Males	1.478 (0.754~2.897)	0.255		

Smoke	1.263 (0.662~2.409)	0.479		

Hypertension	0.509 (0.242~1.068)	0.074		

Diabetes	0.782 (0.371~1.645)	0.516		

Serum creatinine	1.009 (1.006~1.012)	<0.001	1.005 (1.002~1.009)	0.002

CHOL	0.772 (0.563~1.058)	0.107		

Hemoglobin	1.006 (0.989~1.022)	0.508		

Albumin	0.862 (0.811~0.915)	<0.001	1.016 (0.925~1.116)	0.747

lg NT-proBNP	18.632 (7.950~43.669)	<0.001	5.618 (2.082~15.161)	0.001

LVEF	0.977 (0.962~0.993)	0.004	0.989 (0.970~1.009)	0.282

Usage of diuretics	0.719 (0.297~1.739)	0.464		


**Abbreviations:** OR: odds ratio; CHOL: total cholesterol; NT-proBNP: N-terminal B-type natriuretic peptide; LVEF: left ventricle ejection fraction.

On the other hand, univariate logistic regression analysis found that serum calcium was notably related to MACEs (OR: 0.472, 95% CI: 0.373~0.596, p < 0.001). After adjusting for age, serum creatinine, albumin, lg NT-proBNP and LVEF, a lower serum calcium level could be served as a risk factor for MACEs (OR: 0.549, 95% CI: 0.396~0.760, p < 0.001).

**Figure 2:**
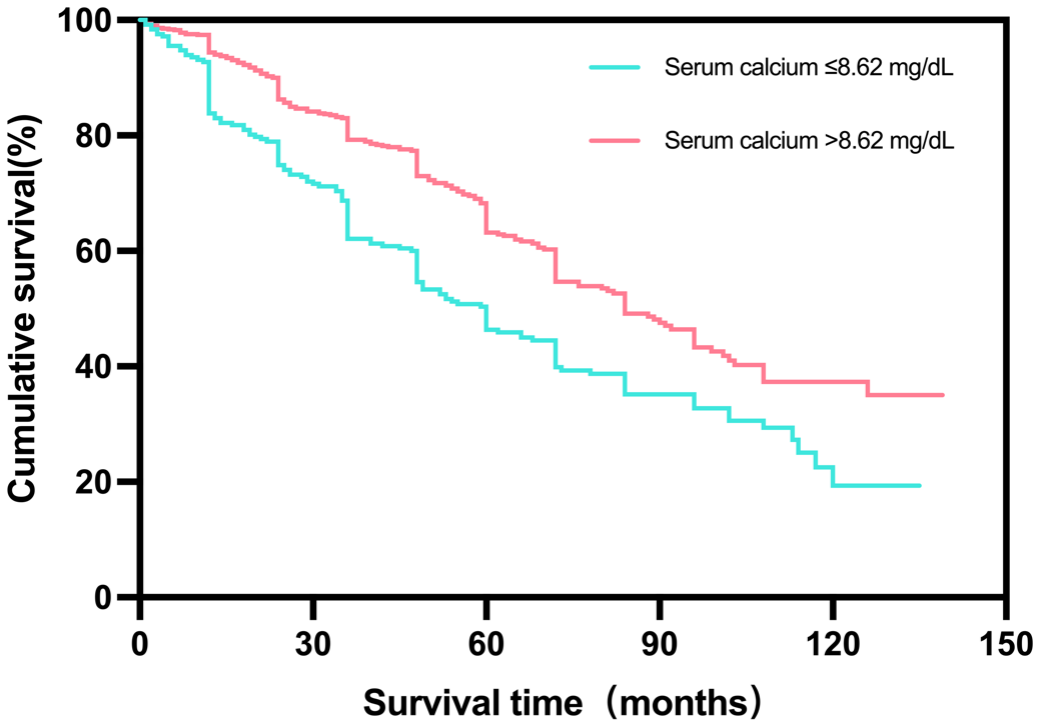
Kaplan-Meier survival curves according to the optimal cutoff value of serum calcium.

The optimal cutoff value of predicting long-term death was close to that for in-hospital mortality. Kaplan–Meier survival estimates indicated that patients with a serum calcium >8.62 mg/dL had a better prognosis than those with a serum calcium ≤8.62 mg/dL (log-rank χ^2^ 40.84, p < 0.001) (Figure 2). Multivariate Cox proportional hazard analysis presented that serum calcium on admission remained a meaningful predictor for long-term mortality after multivariable risk adjustment (HR: 0.708; 95% CI: 0.557~0.901; p = 0.005), meanwhile, a serum calcium ≤8.62 mg/dL was related to a higher proportion of all-cause death (HR: 1.449; 95% CI: 1.115~1.882; p = 0.005) (Table 3).

**Table 3:** Multivariate Cox proportional hazard regression models for long-term mortality.


CLINICAL VARIABLES	HR	95% CI	p-VALUE

Model 1			

Serum calcium, mg/dL	0.708	0.557~0.901	0.005

Age, years	1.038	1.019~1.056	<0.001

Serum creatinine, umol/L	1.004	1.002~1.005	<0.001

Albumin, g/L	1.005	0.977~1.034	0.708

lgNT-proBNP	1.362	1.075~1.725	0.010

LVEF, %	0.977	0.965~0.989	<0.001

Model 2			

Serum calcium ≤ 8.62 mg/dL	1.449	1.115~1.882	0.005

Age, years	1.038	1.02~1.056	<0.001

Serum creatinine, umol/L	1.004	1.003~1.005	<0.001

Albumin, g/L	1.003	0.976~1.03	0.853

lgNT-proBNP	1.333	1.053~1.689	0.017

LVEF, %	0.975	0.963~0.987	<0.001


**Abbreviations:** HR: hazard ratio; CI: confidence interval; LVEF: left ventricular ejection fraction.

## Discussion

In the present study, we selected a relatively large Chinese cohort to investigate the prognostic role of serum calcium in elderly patients with DCM. Those patients are associated with significant mortality of 45% for 10 years, 72% in lower calcium group especially. The results showed that calcium level on admission was significantly related to short- and long-term outcomes and lower level indicated poor prognosis. This is the first study to reveal the connection between serum calcium and clinical outcomes in DCM.

In developing countries, the older population refers to aged 60 years and older, which tend to combine more underlying health problems and present with a more complicated condition [13,14]. Participants in lower calcium group were older and had a larger proportion of male and presented lower rate of survivals, which is consistent with reported study [15]. It is known that the usage of diuretics can affect calcium excretion [16] and there was no statistical difference in its usage between the two groups, as well as in logistic regression analysis. Worsening renal function is known as a significant predictor for poor outcomes in cardiovascular disease [17,18], but the result of collinearity diagnostics analysis did not show collinearity issue between calcium and creatine. Recent accumulating evidence has suggested that increased creatinine is not associated with deleterious prognosis of chronic HF, because general medical treatment including diuretics and ACEIs/ARBs will lead to a rise in creatinine [19]. Additionally, decreased LVEF and elevated value of BNP are confirmed as predictors for poor prognosis in DCM [20], however, in our cohort of patients with DCM, after adjusting for them, serum calcium remained an effective indicator to assess in-hospital and long-term clinical outcomes.

Calcium is one of most abundant elements in human body and plays an essential role in various physiological functions [21]. Half of serum calcium is present in the form of free ions, 40% is combined with albumin and globulins, and approximately 10% is complexed with oxalate, carbonate, and phosphate. Serum calcium is one of the most common laboratory tests and its value is often adjusted by albumin level in the clinic [22]. However, a growing body of evidence suggests that albumin-adjusted calcium may be unreliable for the classification of calcium status in hospitalized patients [23,24]. Therefore, instead of albumin-adjusted calcium, we employed total calcium to explore its prognostic value in patients with DCM.

Calcium ions are the ubiquitous signal transduction molecules in the cells and present an important role in regulating cardiac physiology and electrophysiology. In cardiomyocytes, intracellular calcium concentrations are strictly regulated and are essential determinants in cardiac excitation-contraction coupling [25]. However, when calcium homeostasis is impaired, cardiac electrical and contractile dysfunction will appear and can result in DCM and HF [26]. Similarly, extracellular calcium environment is tightly controlled and is jointly influenced by renal excretion, intestinal absorption, and bone remodeling [27]. Before this, hypocalcemia has been shown to be associated with a significant increase in all-cause mortality in patients with cardiovascular disease [28,29].

Interestingly, in the general population, lower serum calcium level is also an independent risk factor for sudden cardiac arrest [30]. The underlying mechanism by which lower serum calcium level may lead to increased risk of death is unclear. It may involve changes in the cardiac electrophysiological function because calcium influx through the L-type calcium channel may be decreased when serum calcium concentration is lower, resulting in reduced depolarization and a shortened cardiac action potential, which will affect the cardiac contractility [31]. However, the specific pathophysiology remains to be elucidated and still need further research.

### Study limitations

This was a retrospective study with some limitations. First, this was a single-center study, and the number of patients with baseline reduced calcium level was relatively small. We will recruit more admitted patients to expand the database. Second, potential confounding factors may have affected the results due to the inherent flaws in the study design, even after the adjusted analysis. Third, the specific mechanism by which serum calcium influence the prognosis of patients with DCM has not been verified in this study.

## Conclusions

Univariate and multivariate logistic regression analyses showed that serum calcium was independently associated with in-hospital mortality and long-term MACEs in patients with DCM. Serum calcium level could be served as a simple and affordable tool to evaluate prognosis in DCM patients. Future studies are warranted to elucidate the underlying mechanisms between the lower calcium and poor clinical outcomes of patients with DCM.

## Data Accessibility Statement

The datasets generated during and/or analyzed during the current study are not publicly available due to privacy or ethical restrictions but are available from the corresponding author on reasonable request.
